# Supplementary material for: Antioxidant and Antimicrobial Evaluations of Moringa oleifera Lam Leaves Extract and Isolated Compounds
Source: Molecules. 2023 Jan 16;28(2):899. doi: 10.3390/molecules28020899 (PMC9866898; doi:10.3390/molecules28020899)

STRUCTURAL CHARATERIZATION AND ELUCIDATION OF COMPOUND E1

HPLC-PDA OF COMPOUND E1

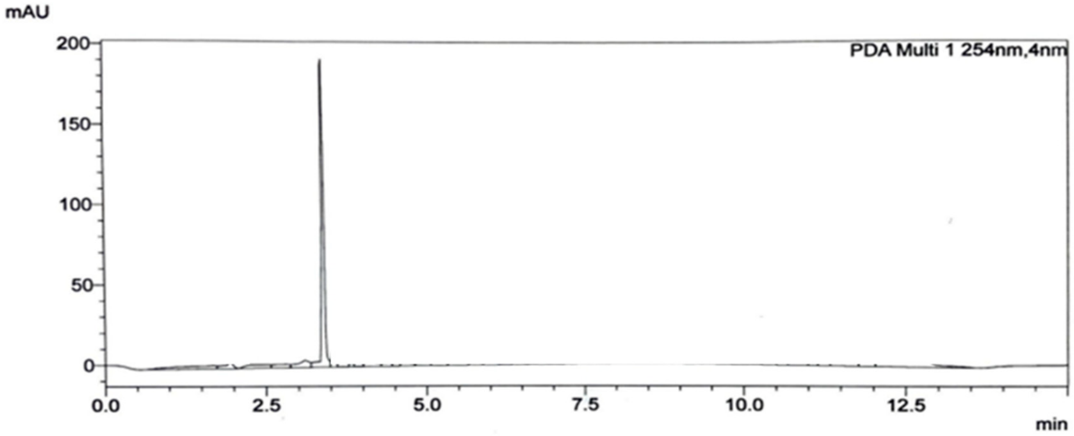

FigureS1: HPLC-PDA OF COMPOUND E1

UPLC-MS OF E1

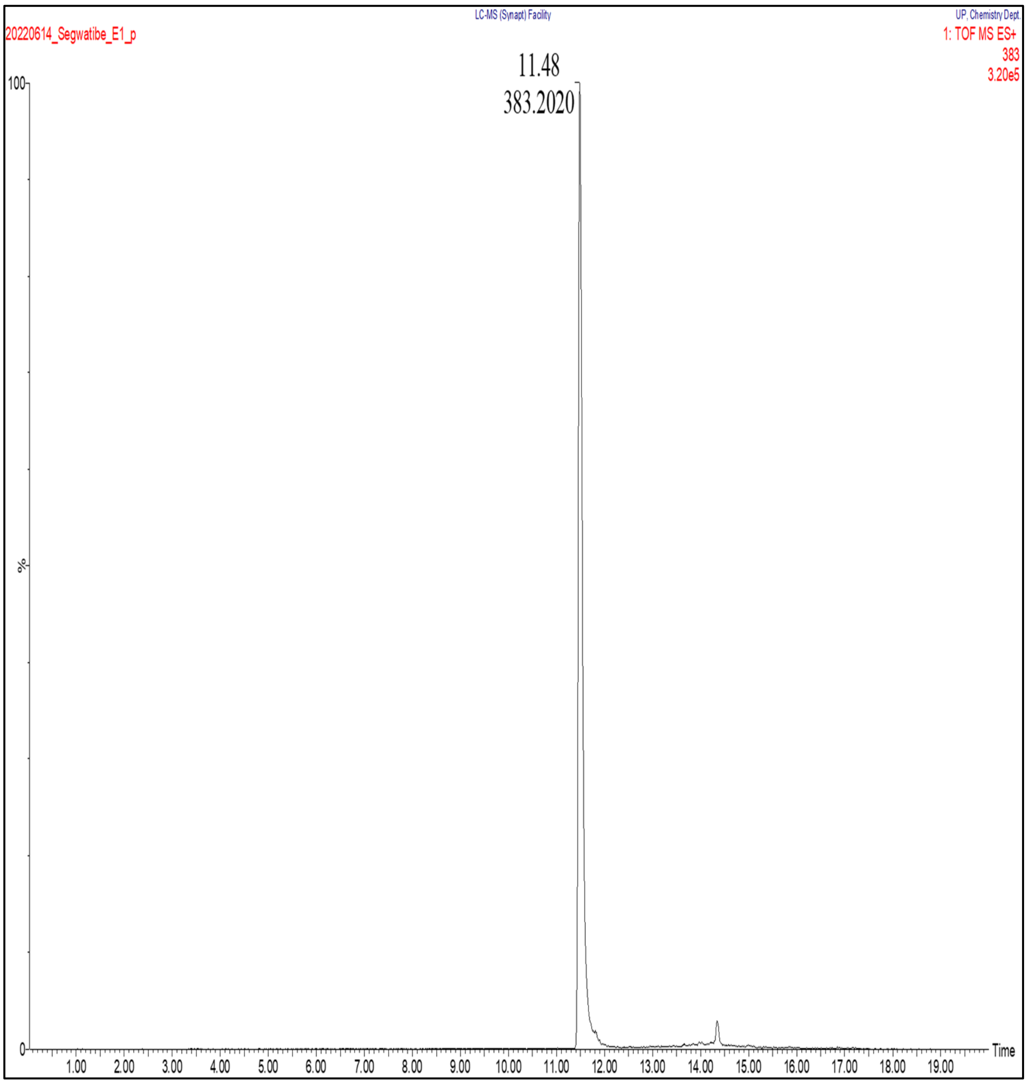

FigureS2: High resolution UPLC-MS of E1 indicating its purity M/z ratio at 383.202.

MS FRAGMENTS OF E1

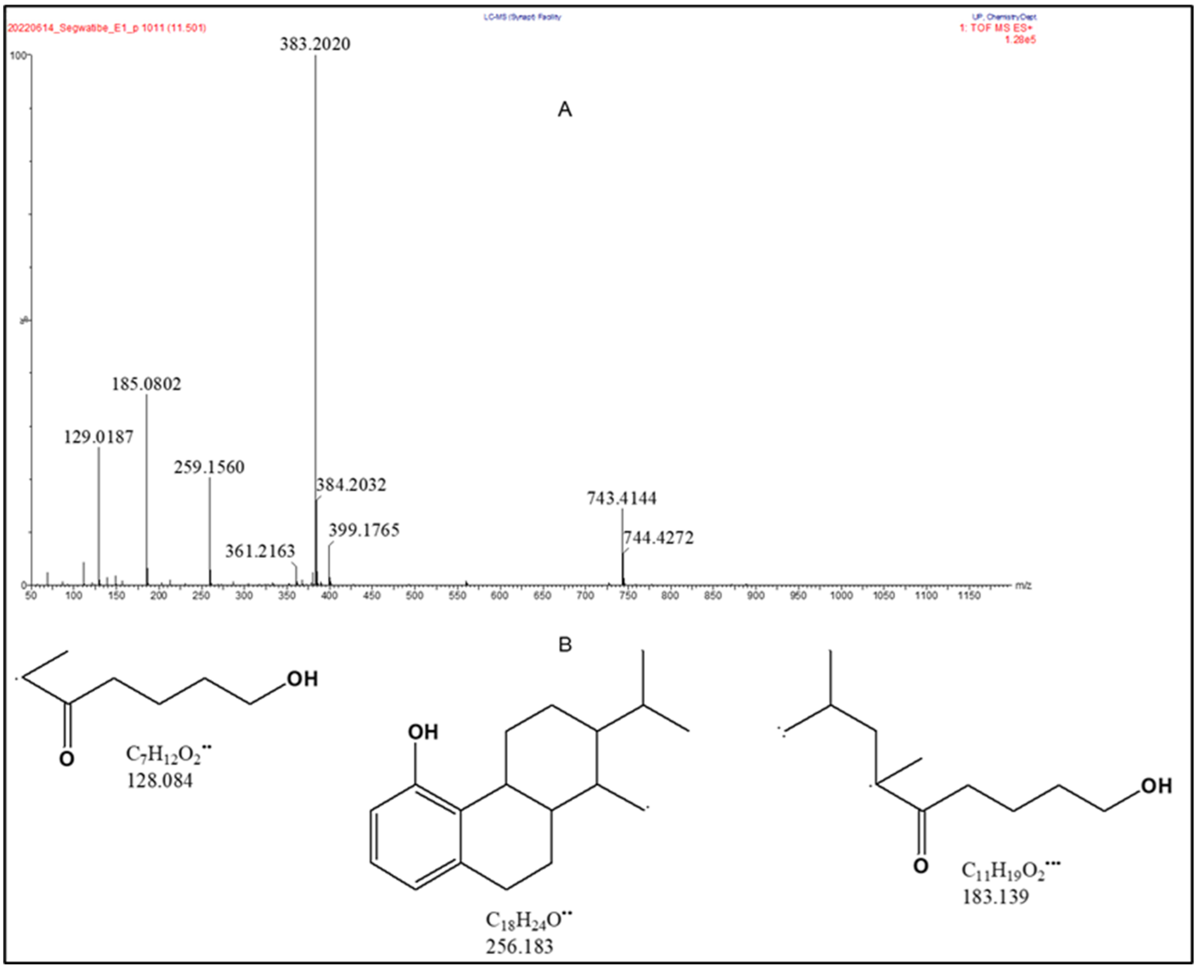

FigureS3: MS FRAGMENTS OF E1

PROTON NMR OF E1

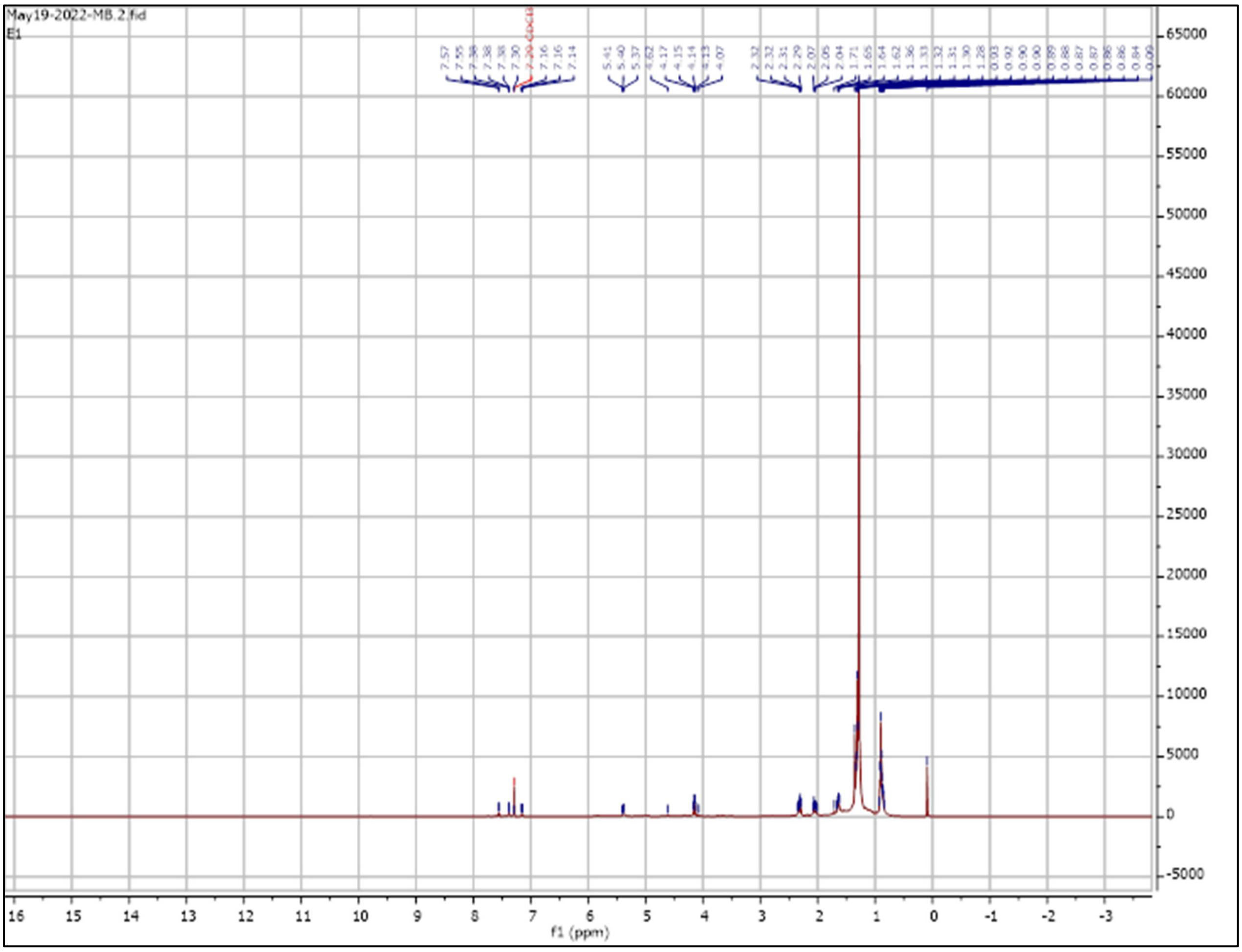

FigureS4 PROTON NMR OF E1

CARBON -13 NMR OF E1

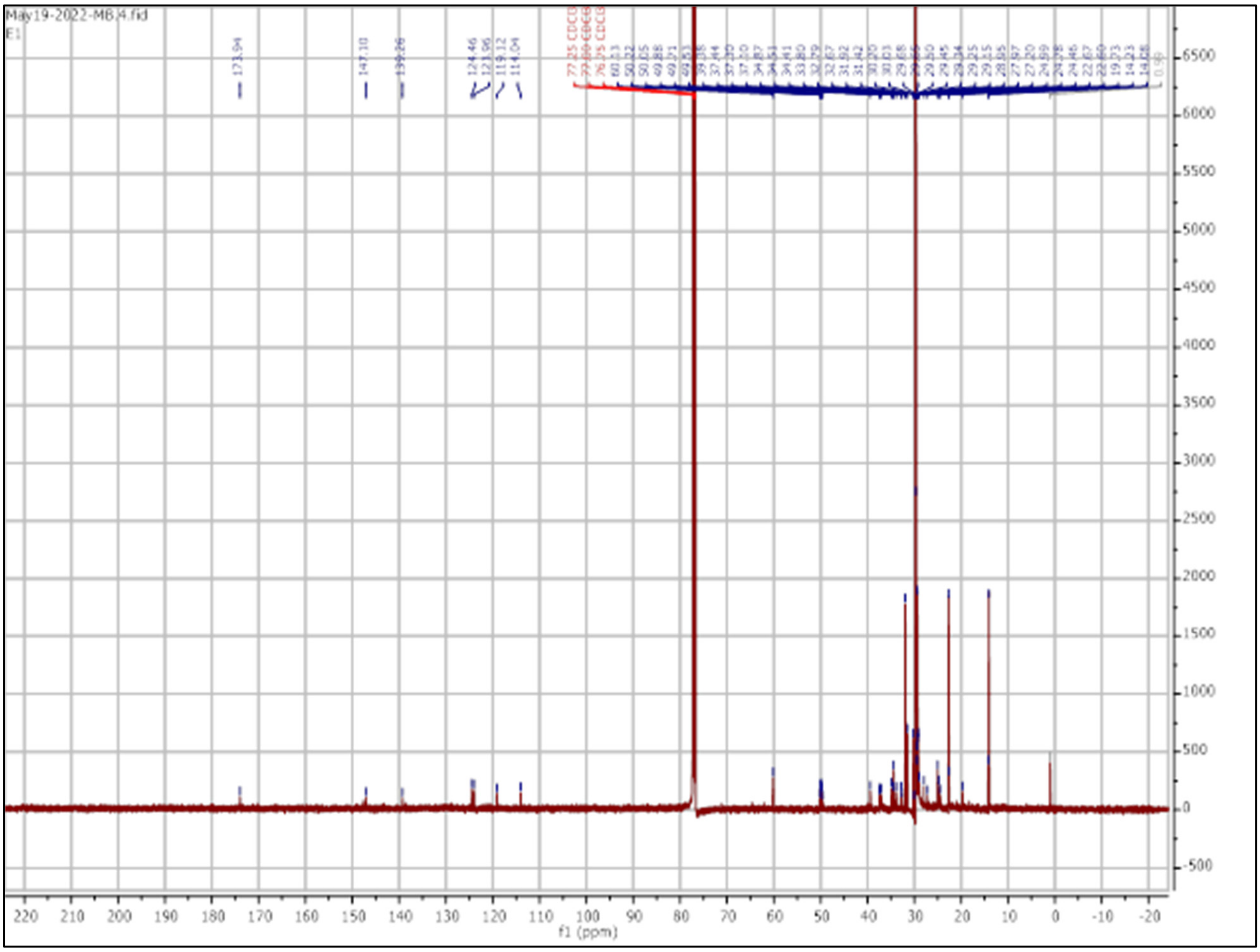

Figure S5 CARBON -13 NMR OF E1

COSY 2D NMR OF E1

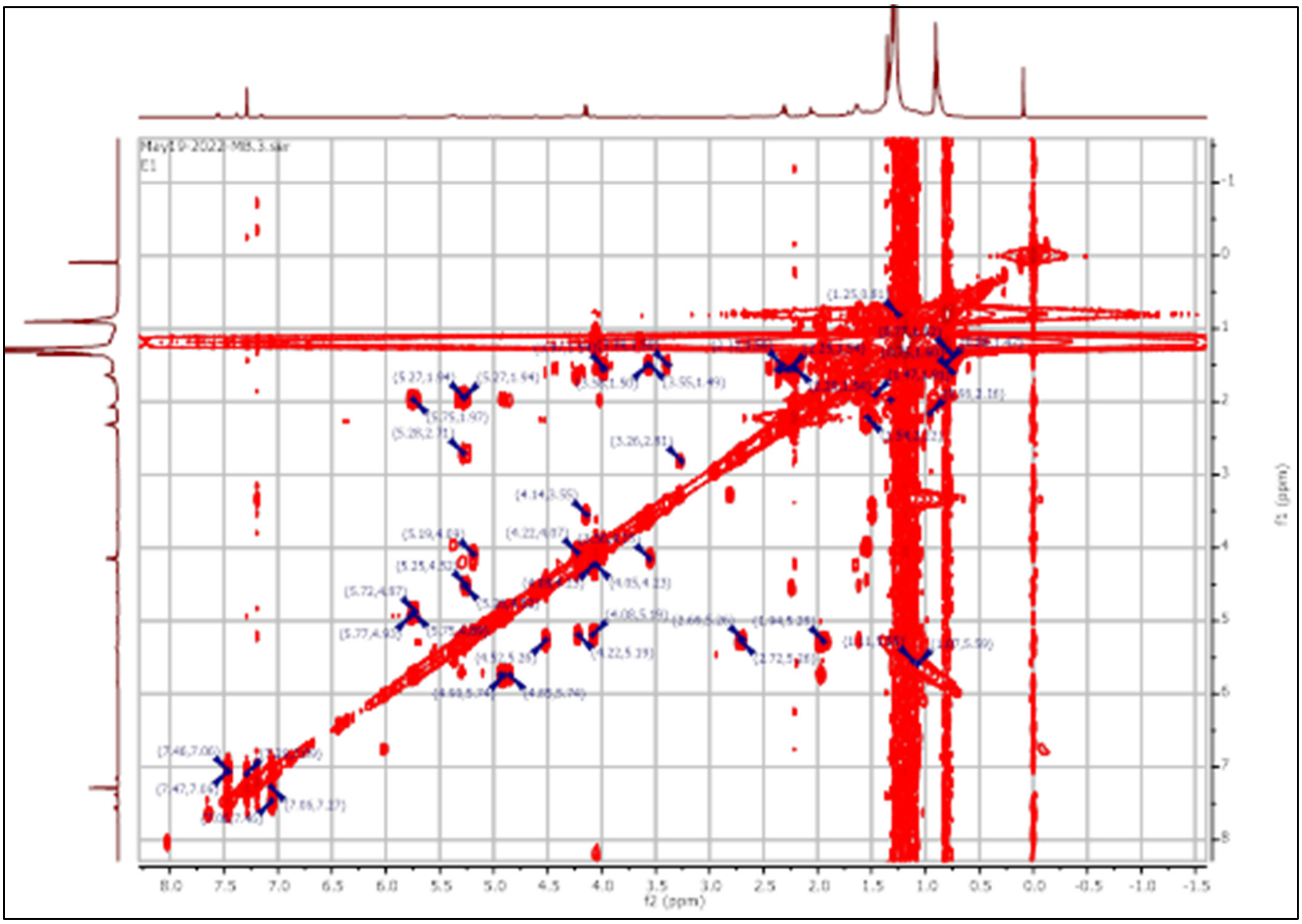

Figure S6 COSY 2D NMR OF E1

DEPT NMR OF E1

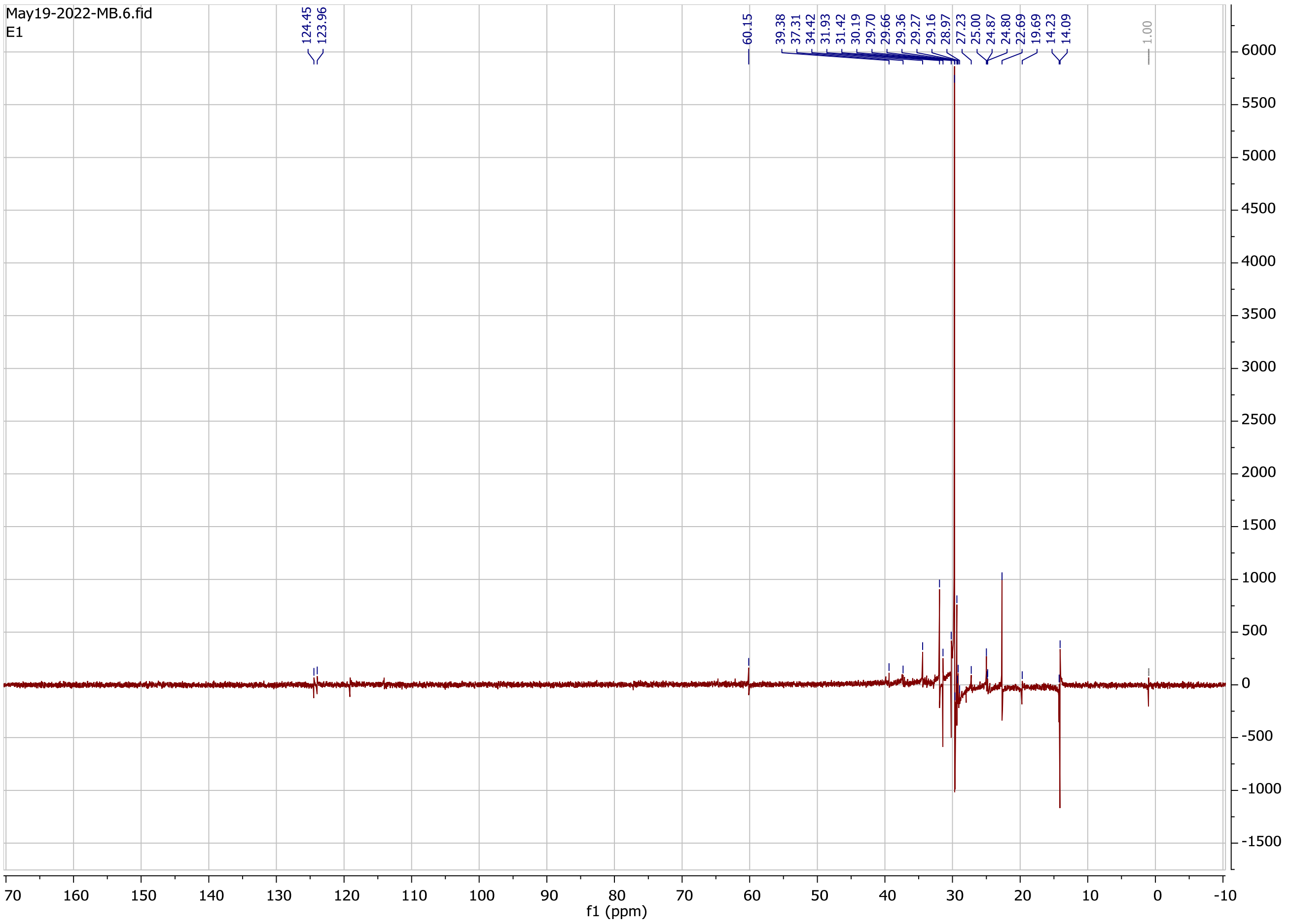

Figure S7 DEPT NMR OF E1

HSQC 2D NMR OF E1

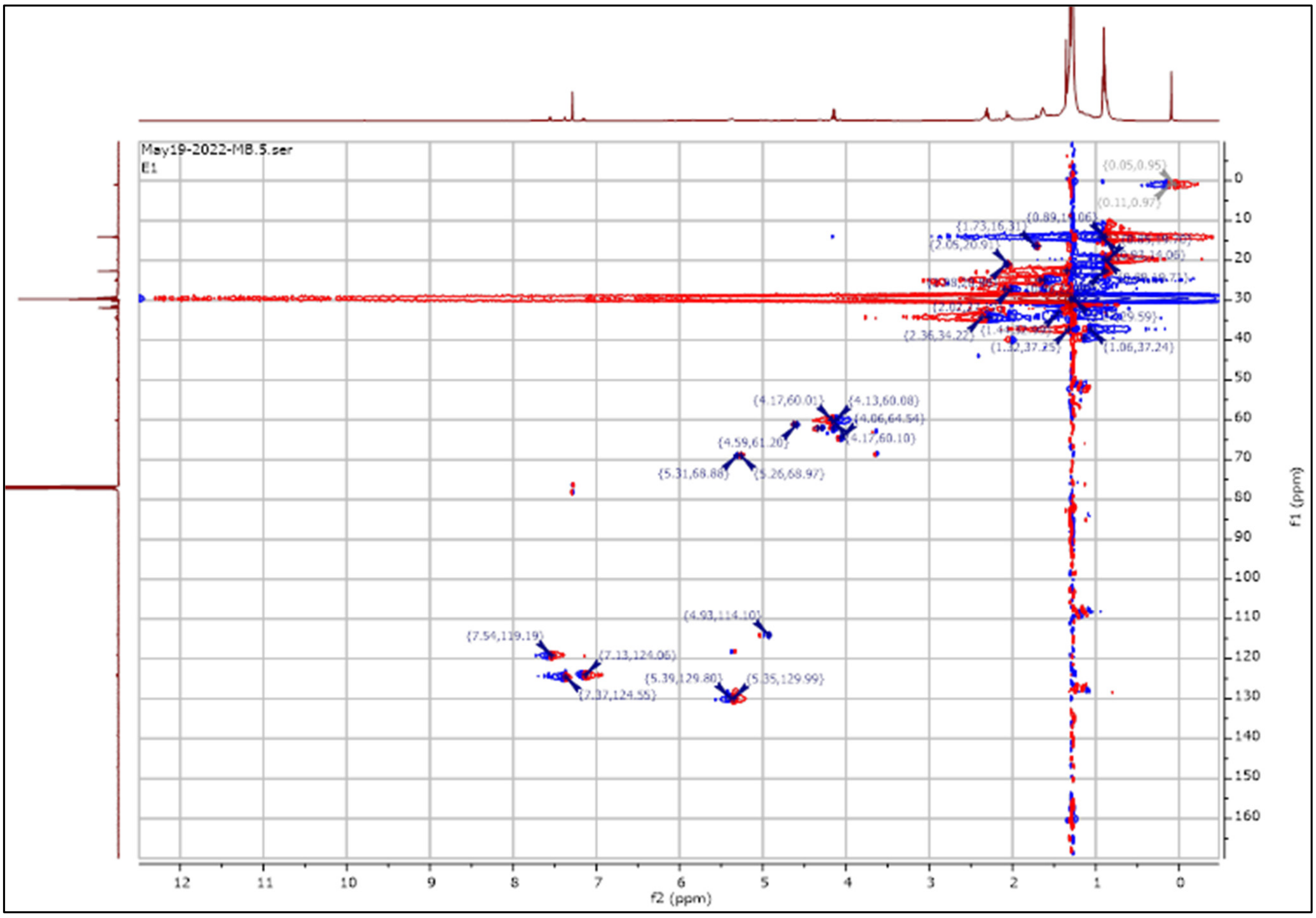

Figure S8 HSQC 2D NMR OF E1

HMBC 2D NMR OF E1

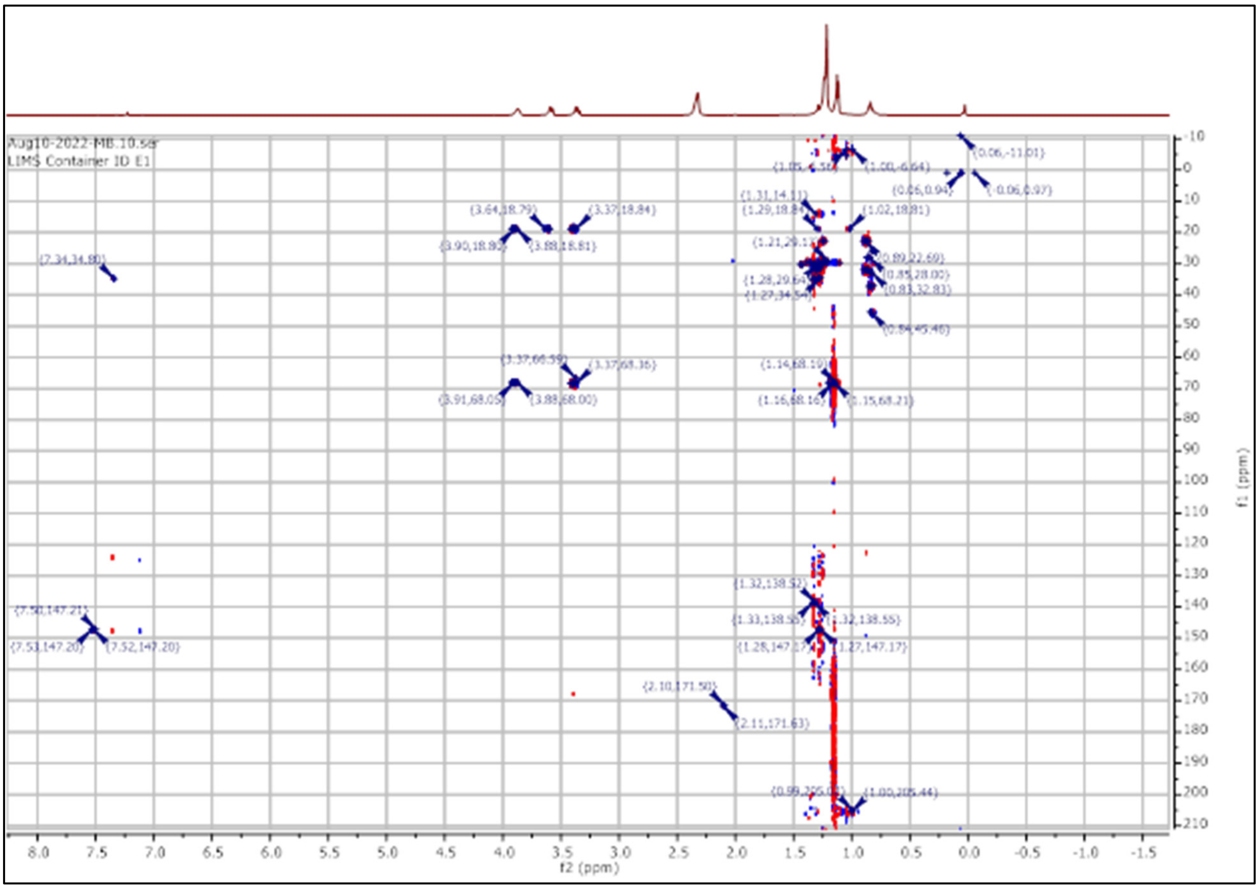

Figure S9 HMBC 2D NMR OF E1

STRUCTURL CHARATERIZATION AND ELUCIDATION OF COMPOUND E3

Figure S10 HPLC-PDA OF COMPOUND E3

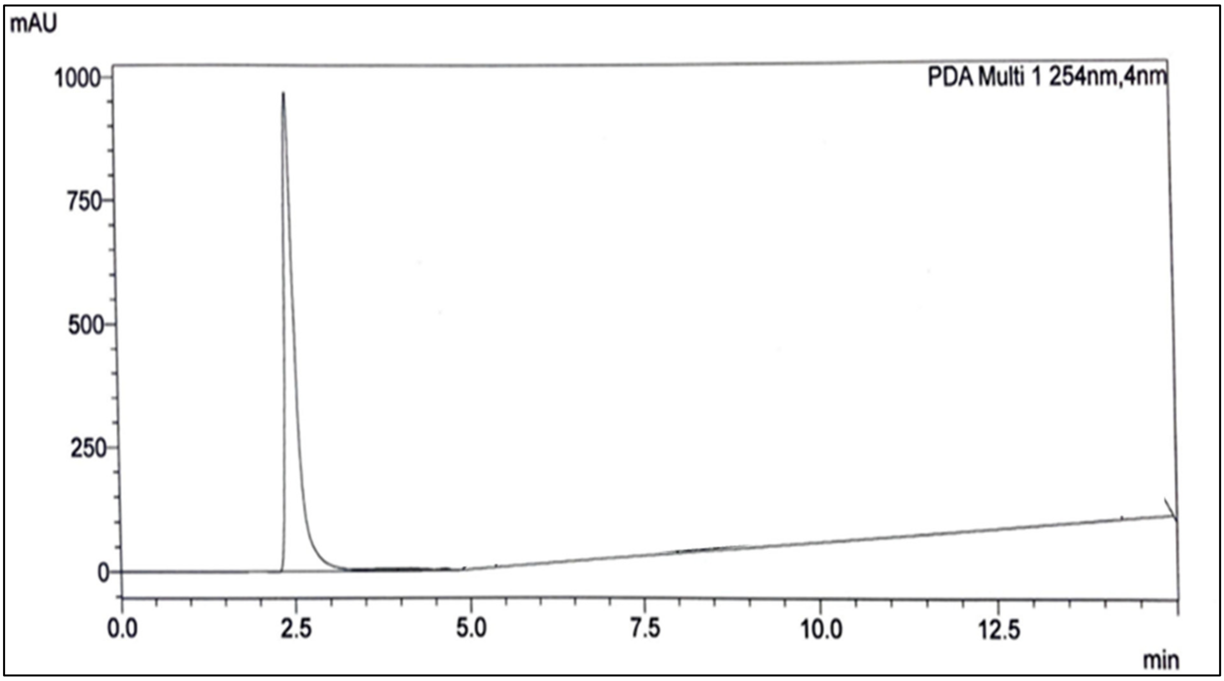

Figure S11 **ABSORBANCE MAXIMA (λ_{MAX}) OF COMPOUND E3**

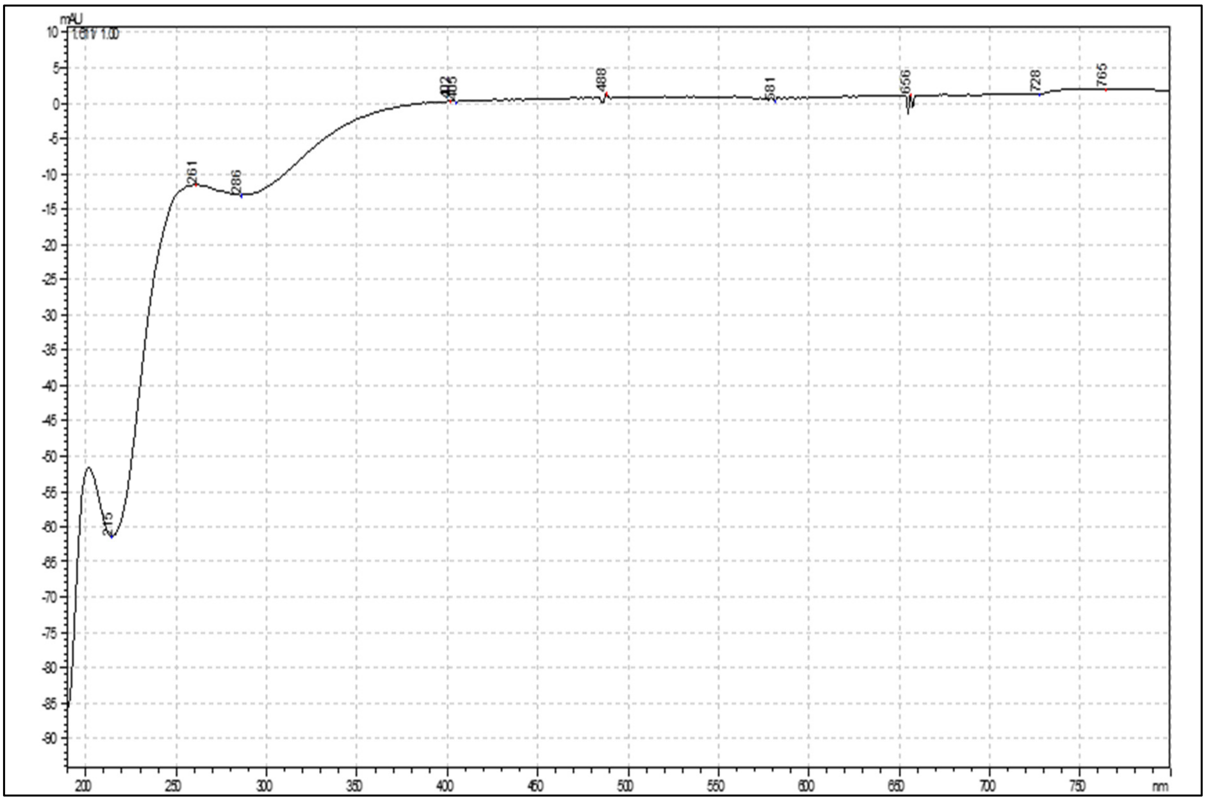

Figure S12 UPLC-MS OF COMPOUND E3

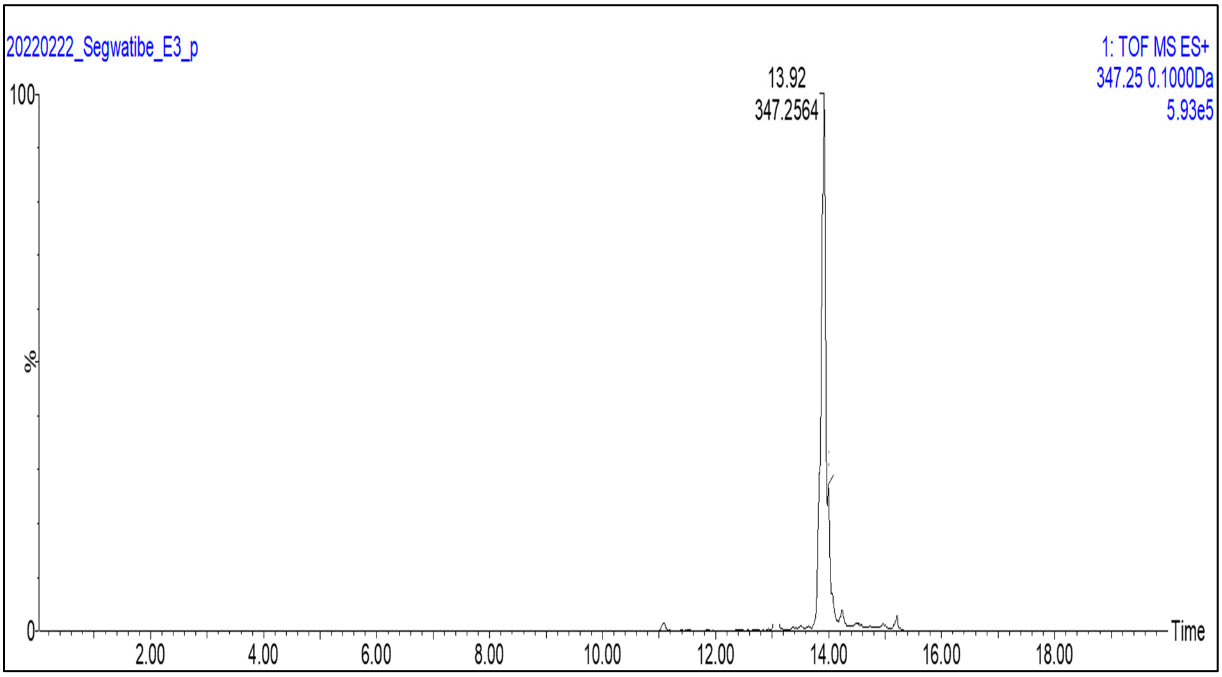

Figure S13 FRAGMENTATION PATTERN OF E3

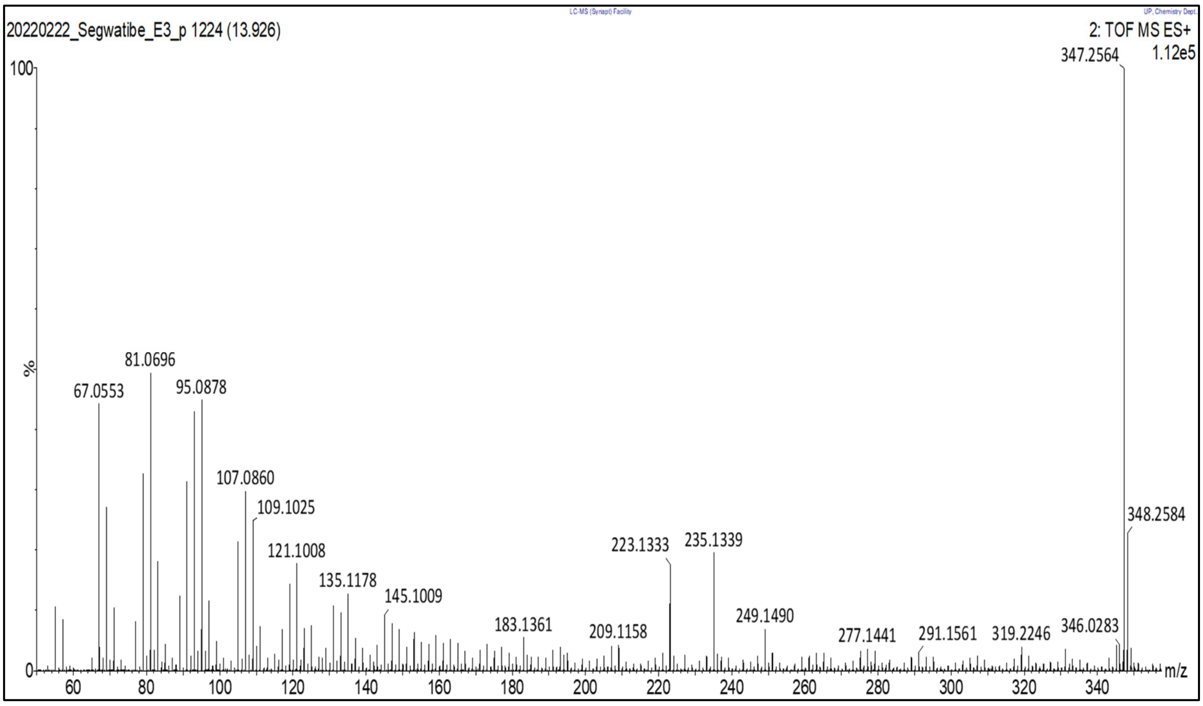

.

Figure S14 CARBON-13 NMR OF COMPOUND E3

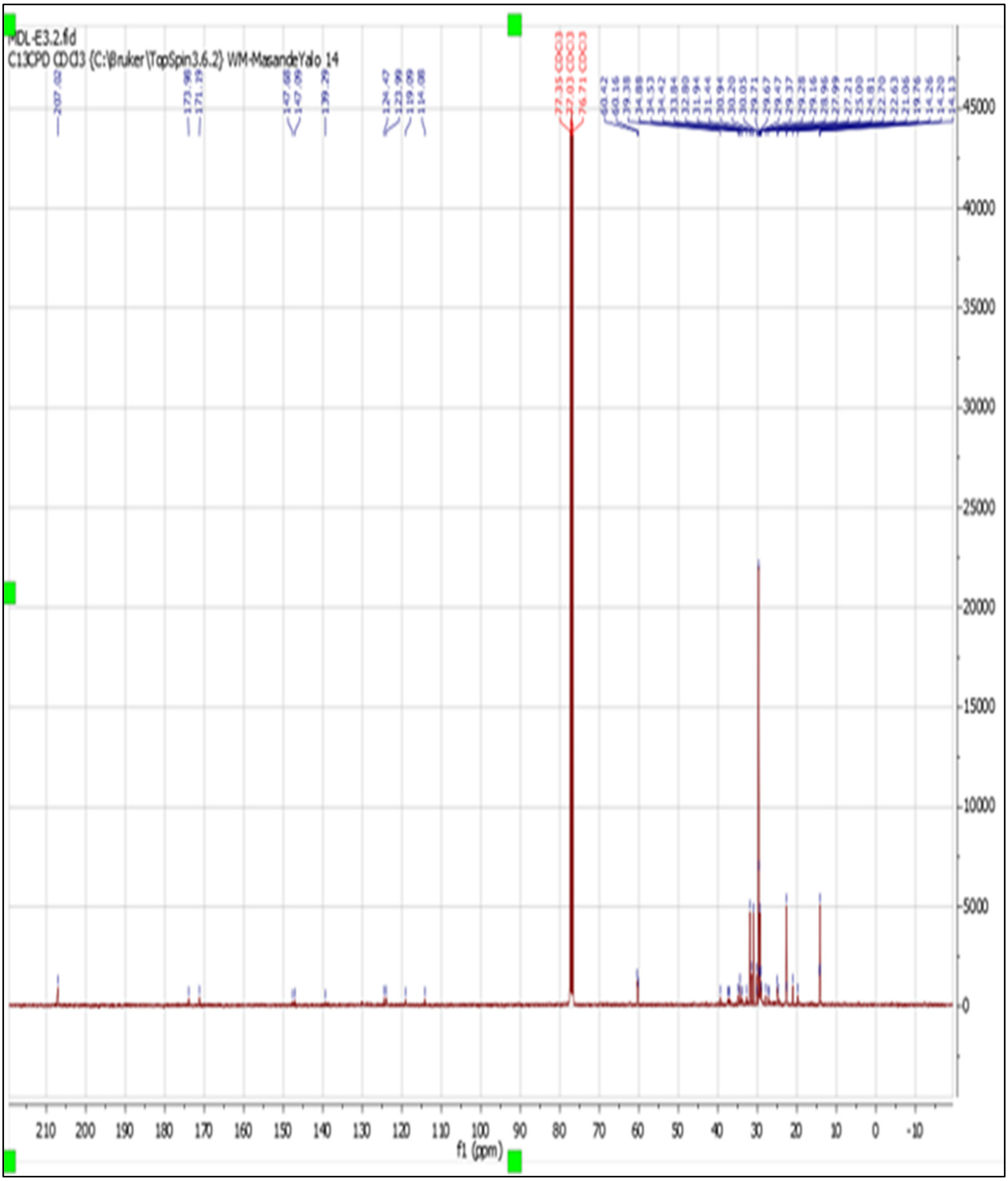

Figure S14 **H-1 NMR OF COMPOUND E3**

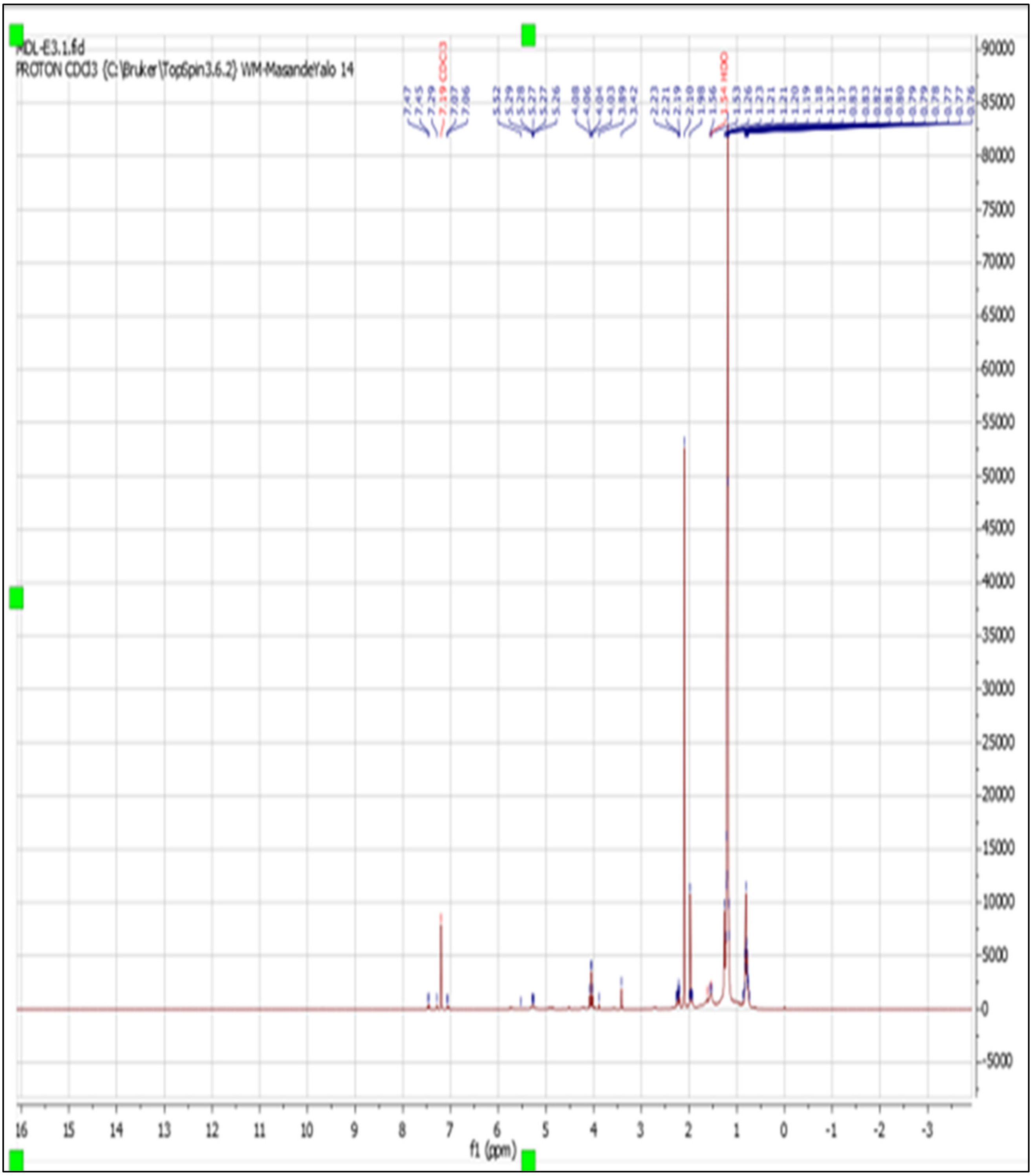

Figure S15 DEPT NMR OF COMPOUND E3

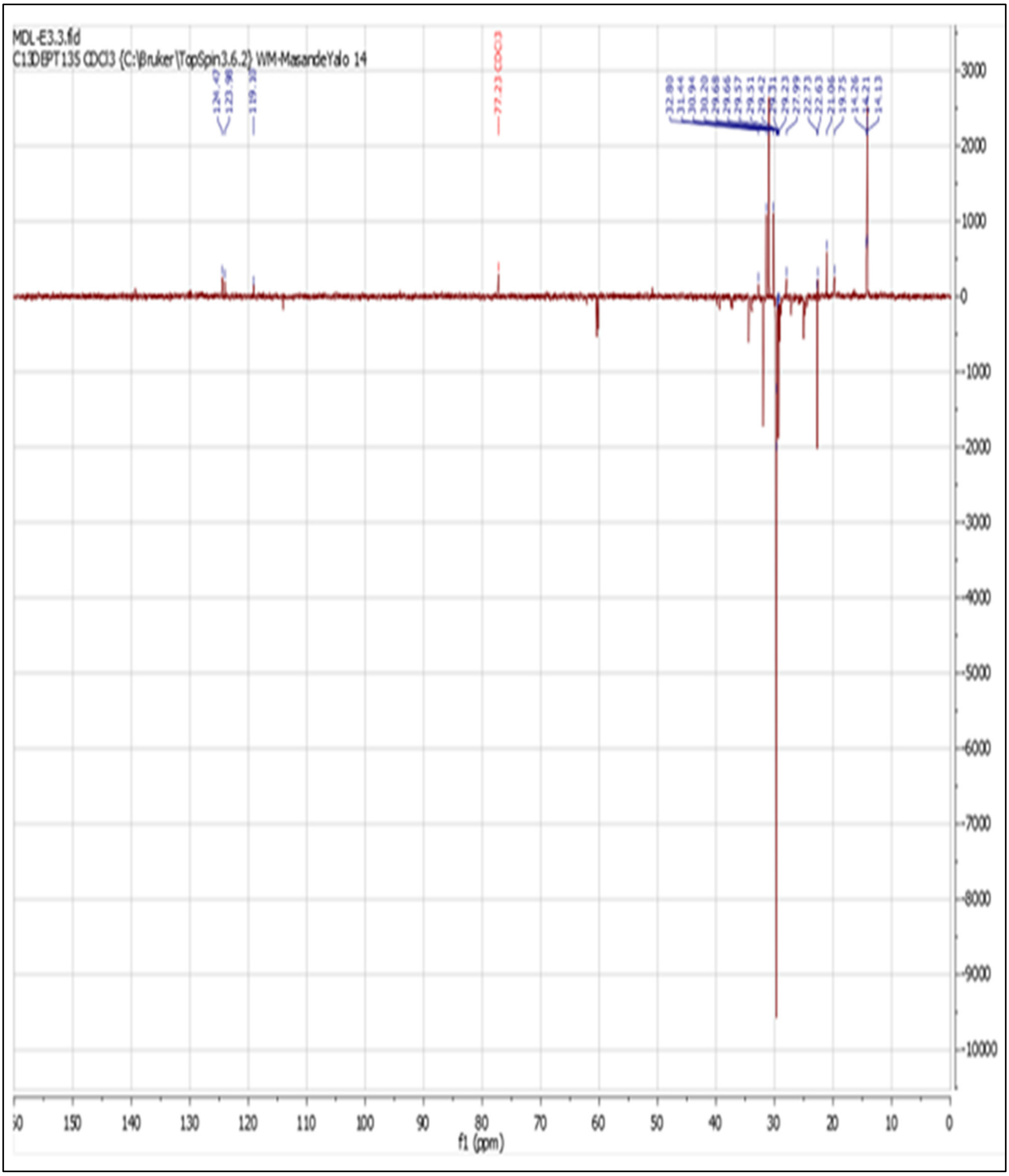

Figure S16 COSY NMR OF COMPOUND E3

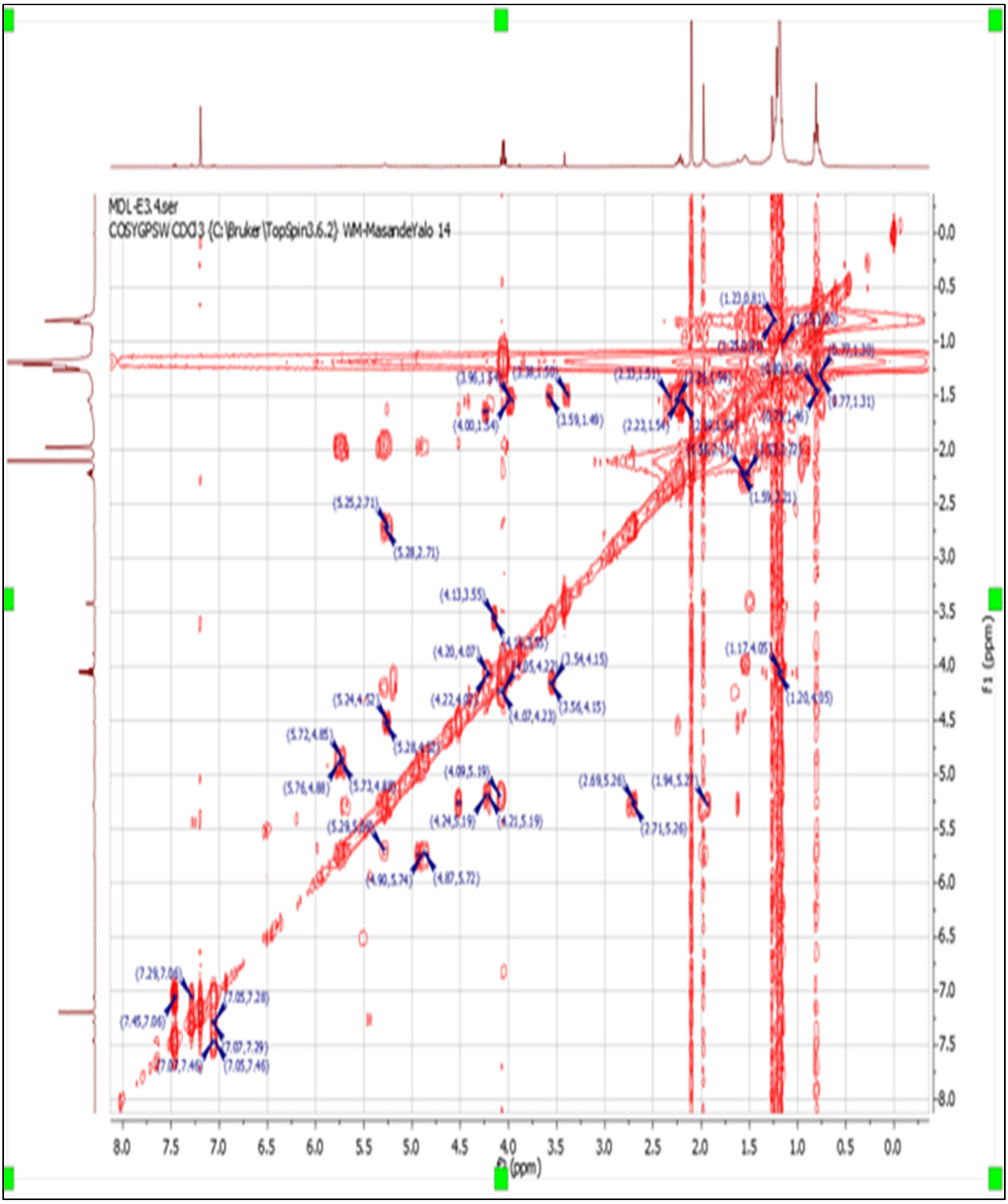

Figure S17 HSQC NMR OF COMPOUND E3

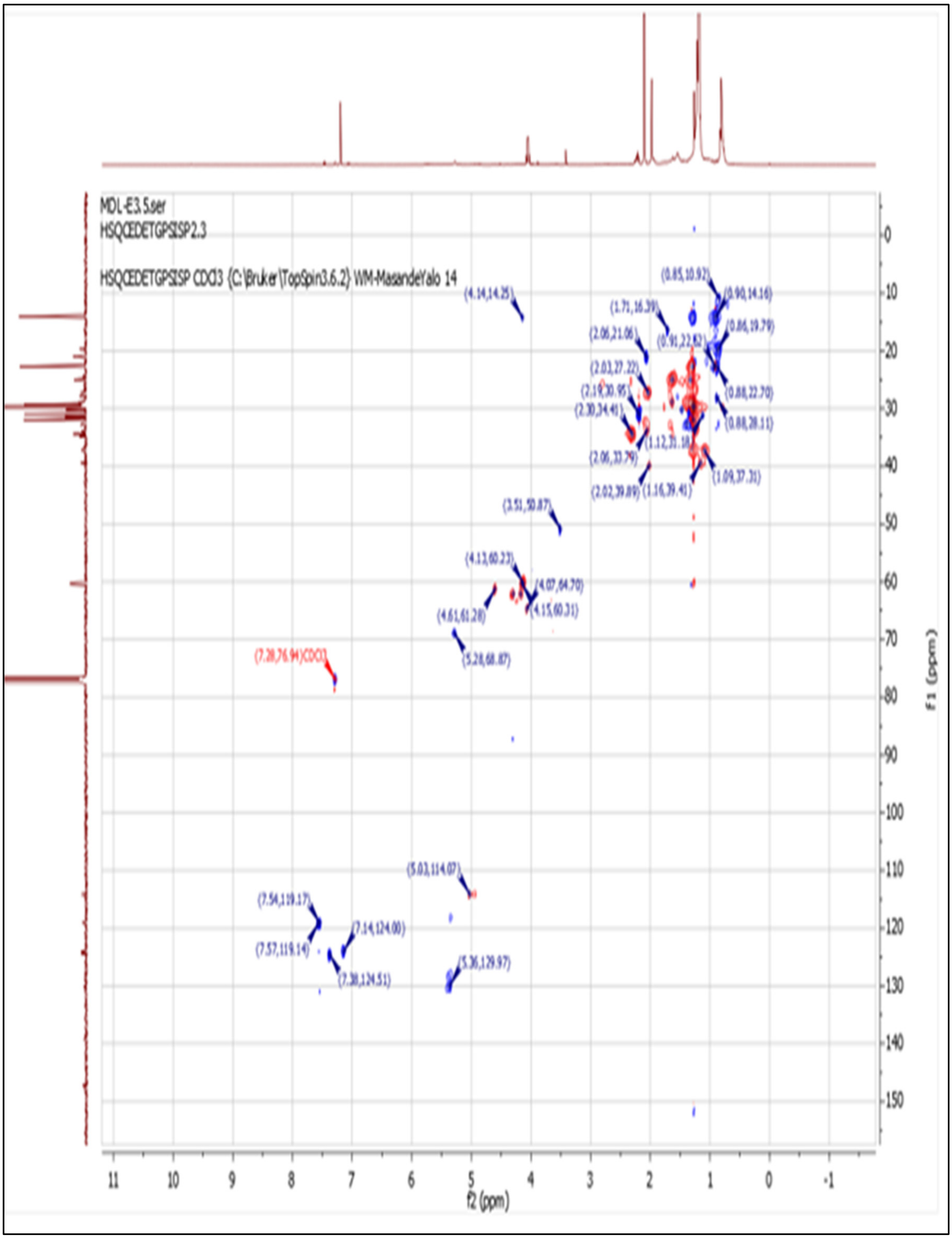

Figure S18 **HMBC NMR OF E3**

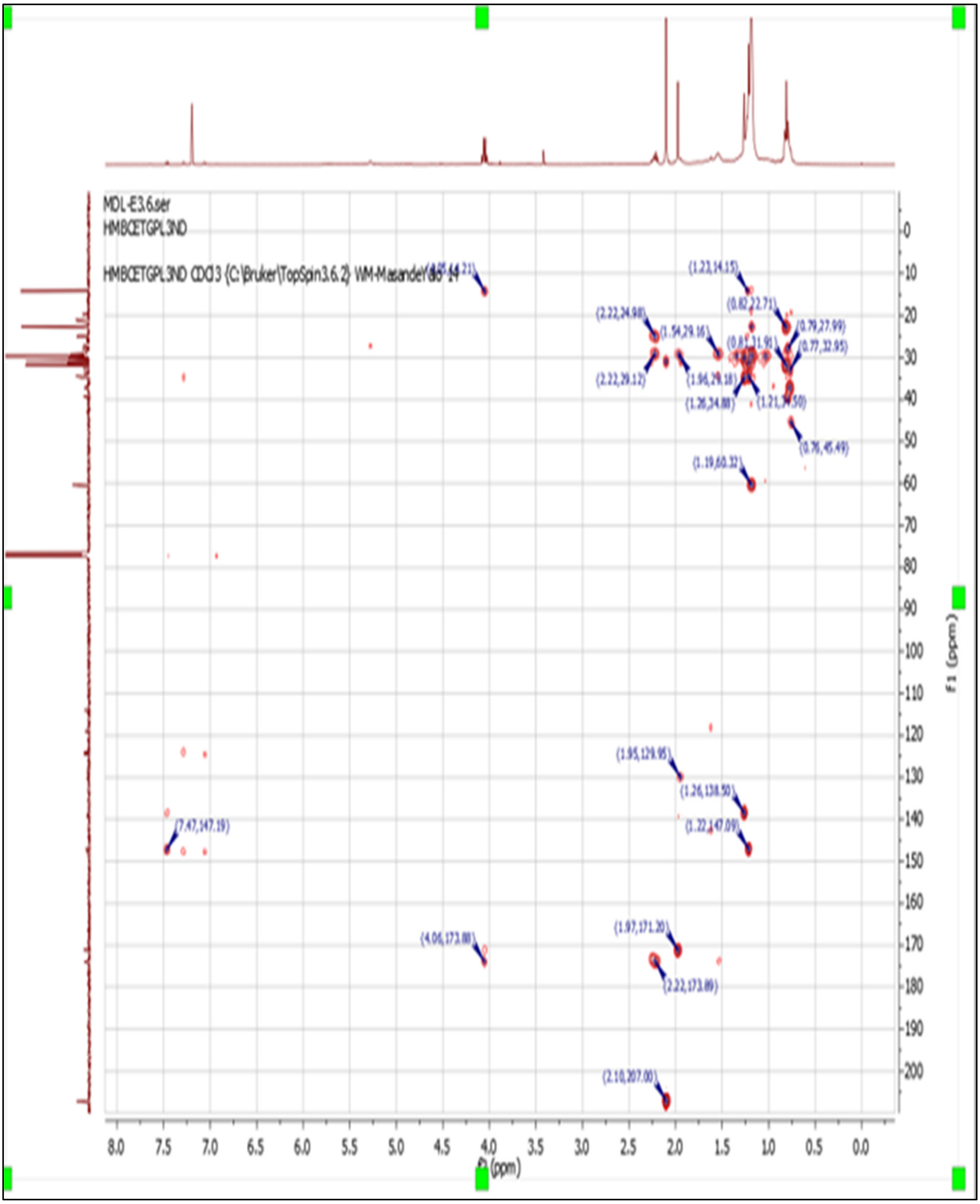

STRUCTURAL CHARATERIZATION AND ELUCIDATION OF COMPOUND Rα

Figure S19 ABBSORBANCE MAXIMA (λ_{MAX}) OF COMPOUND Rα

The importance of absorbance maxima (λ_{MAX}) of Rα, its 196 nm. As shown in below other possible absorbance of Rα appeared at 251 nm and 261 nm.

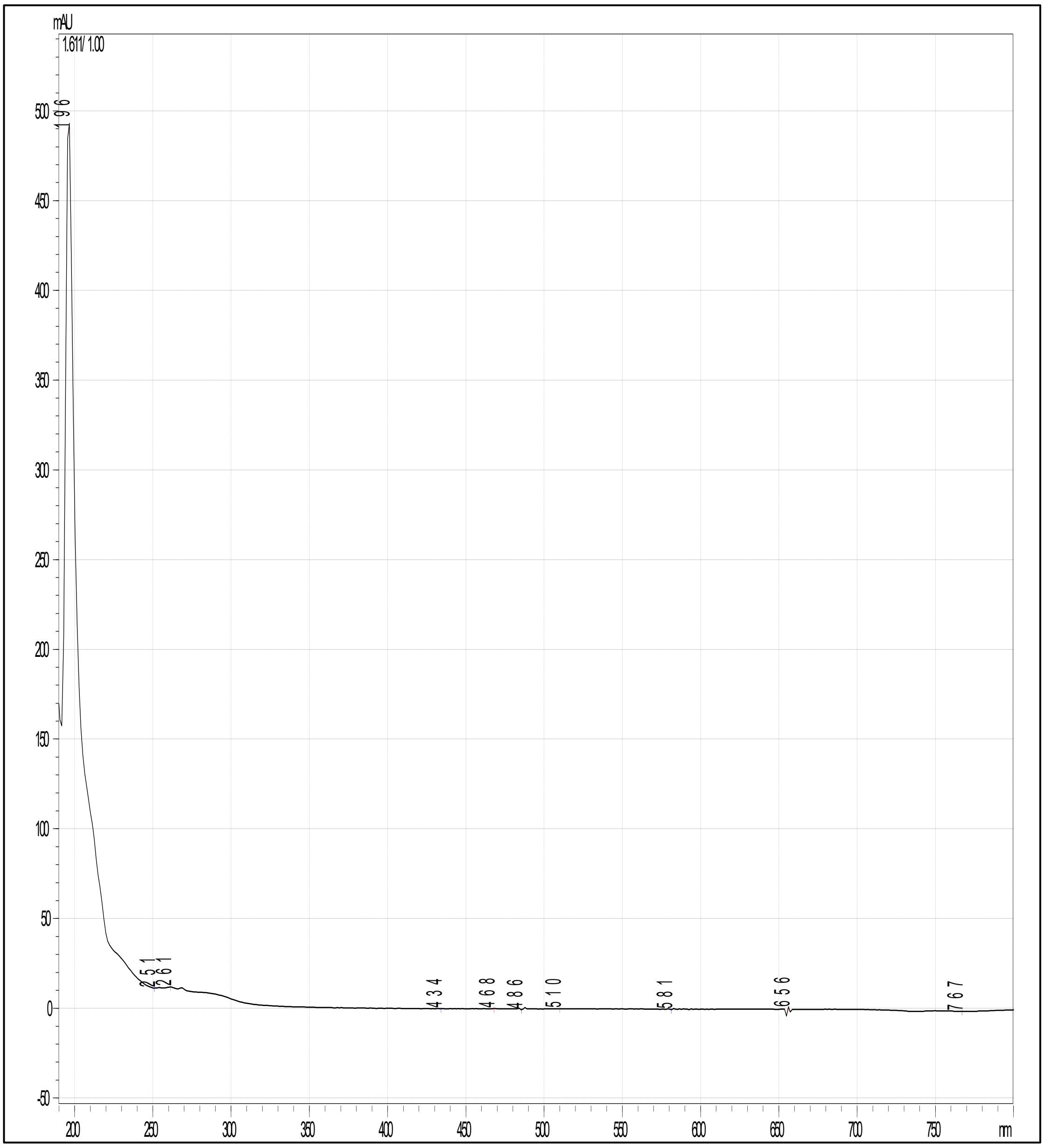

Figure S20 **UPLC-MS OF COMPOUND Rα**

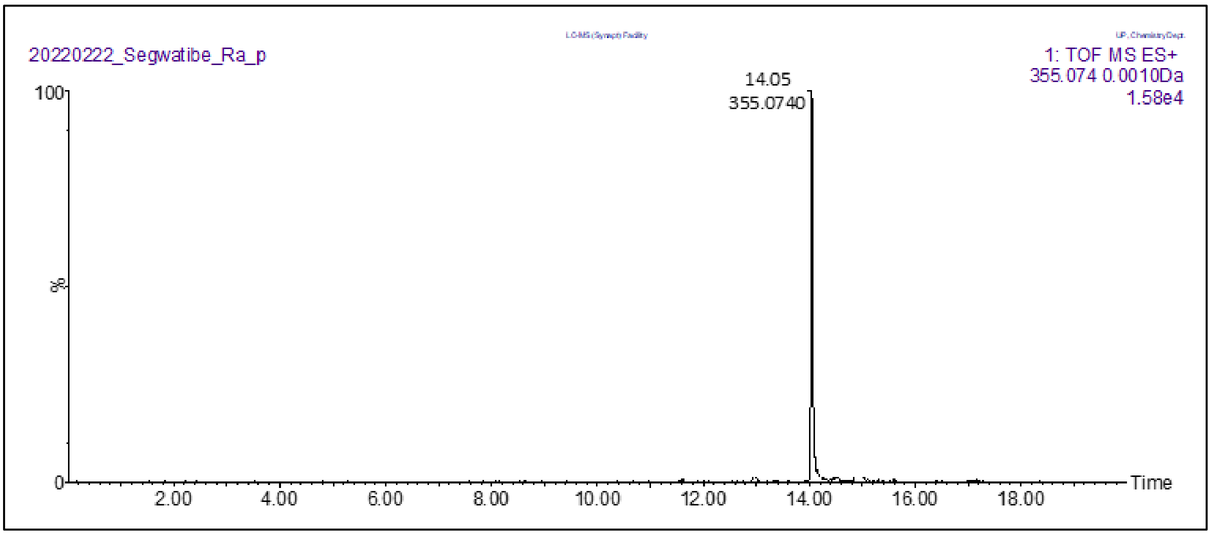

Figure S21 **UPLC-MS FRAGMENTATION OF COMPOUND Rα**

As for the fragmentation pattern of Rα, the major daughter ion was 266. The other fragments were 281, 250, 207, 191, 147, 133, 89 and1nd 73.

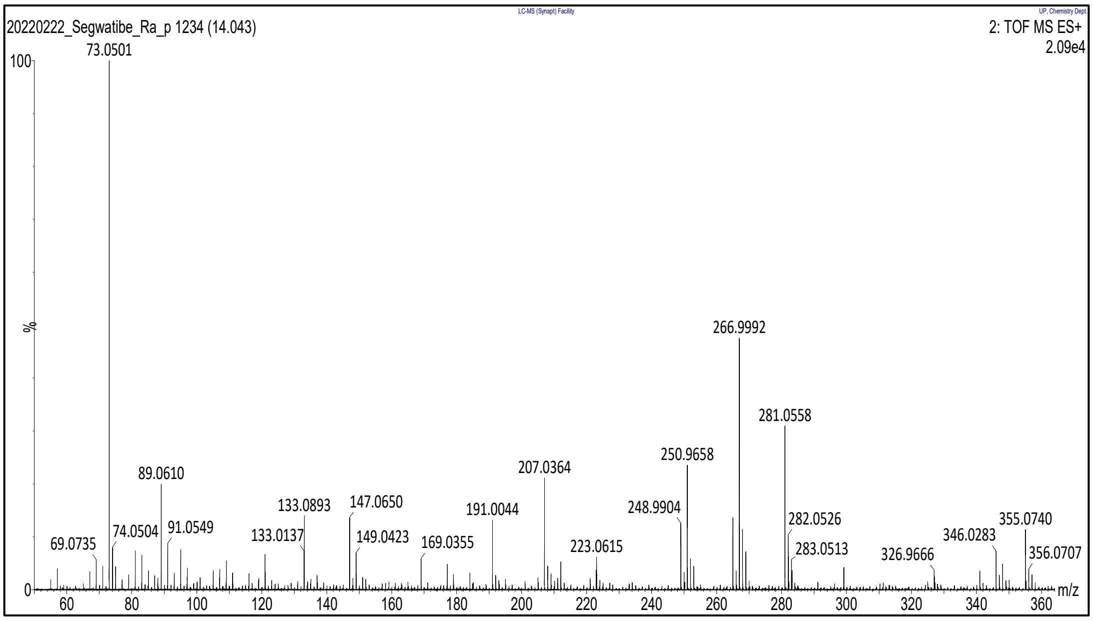

Figure S22 CARBON-13 NMR OF COMPOUND Rα

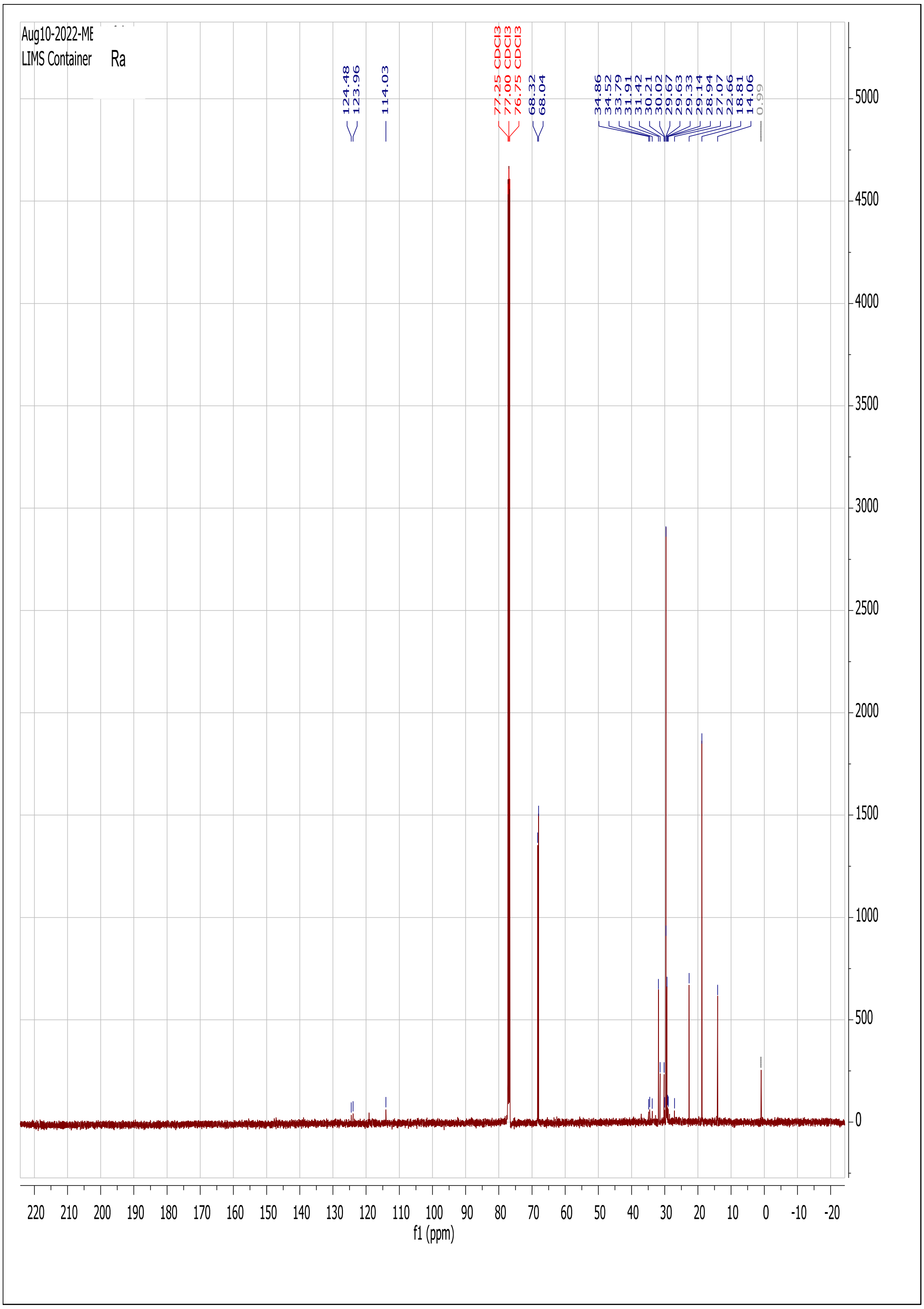

Figure S23 PROTON NMR EXPERIMENT OF Rα

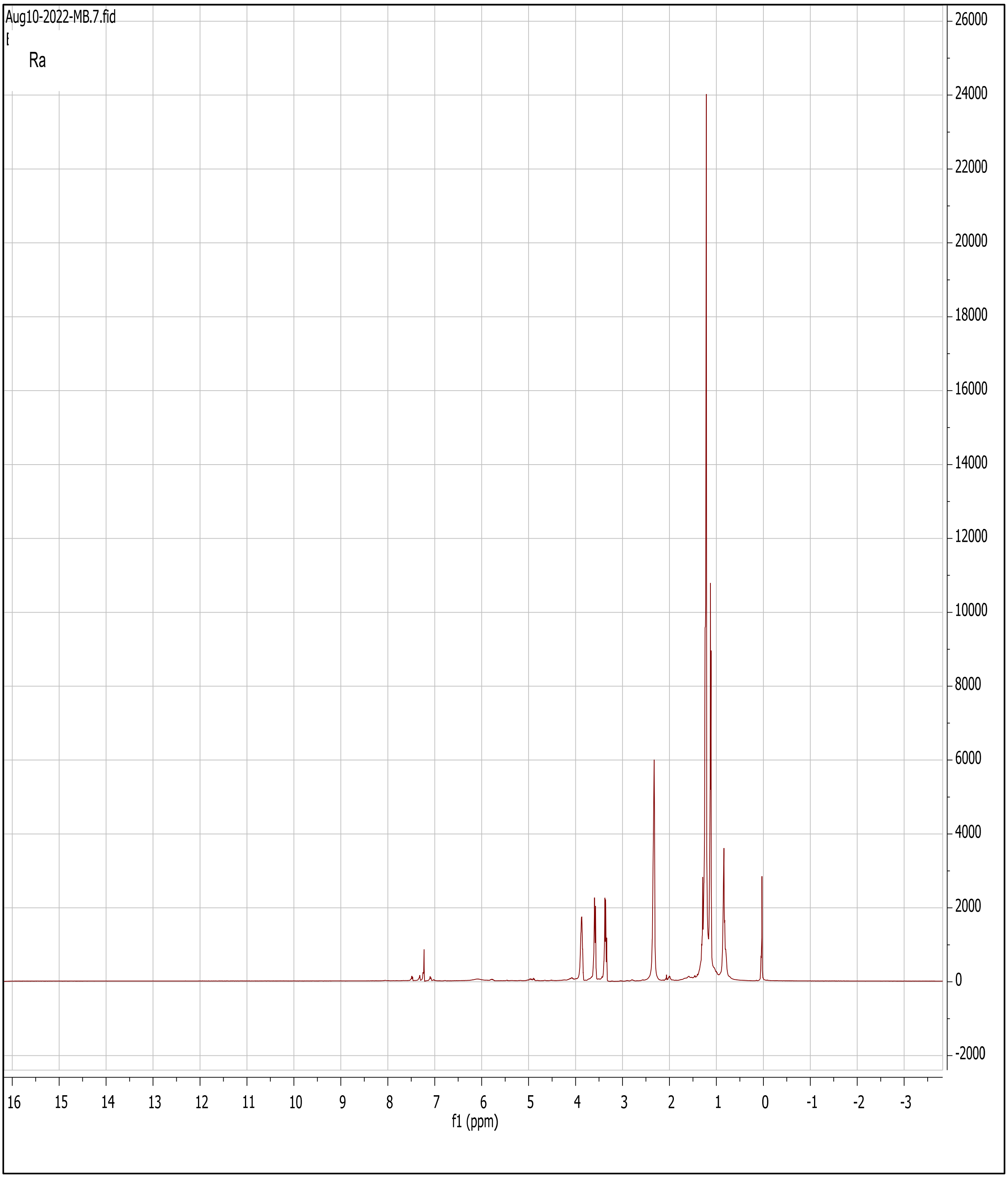

Figure S24 COSY 2D NMR OF Ra

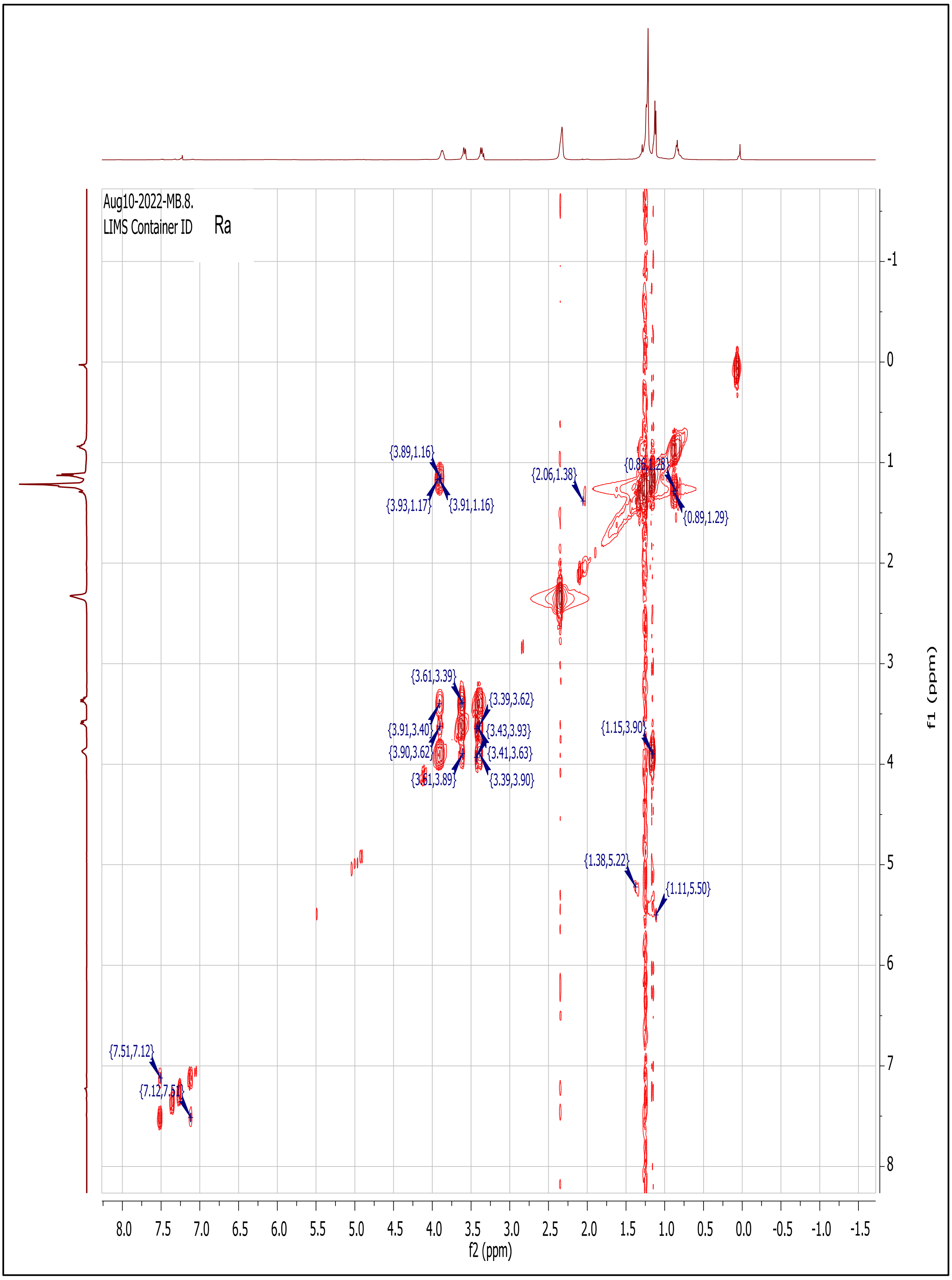

Figure S25 HSQC 2D NMR OF Ra

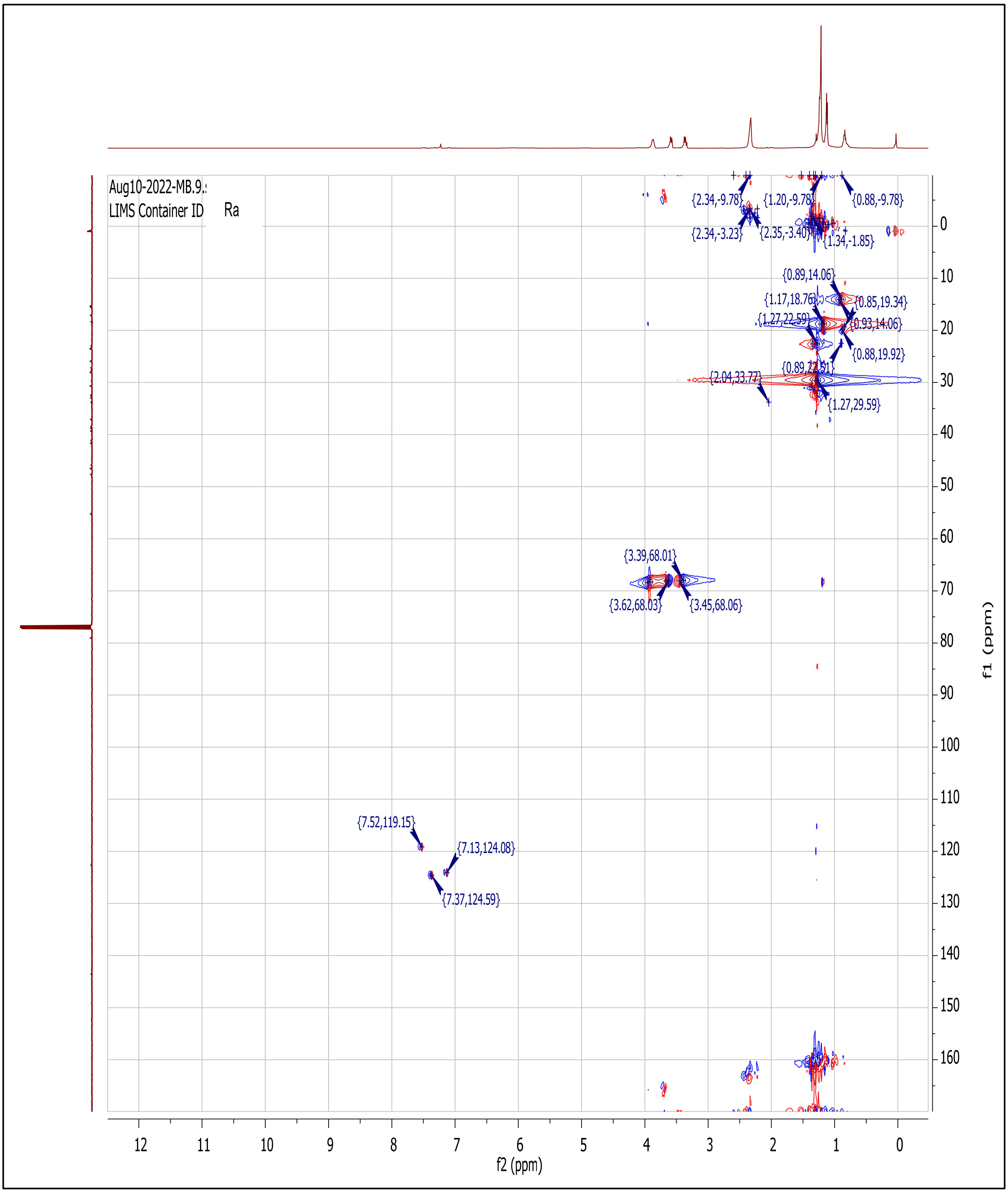

Figure S26 HMBC 2D NMR OF Ra

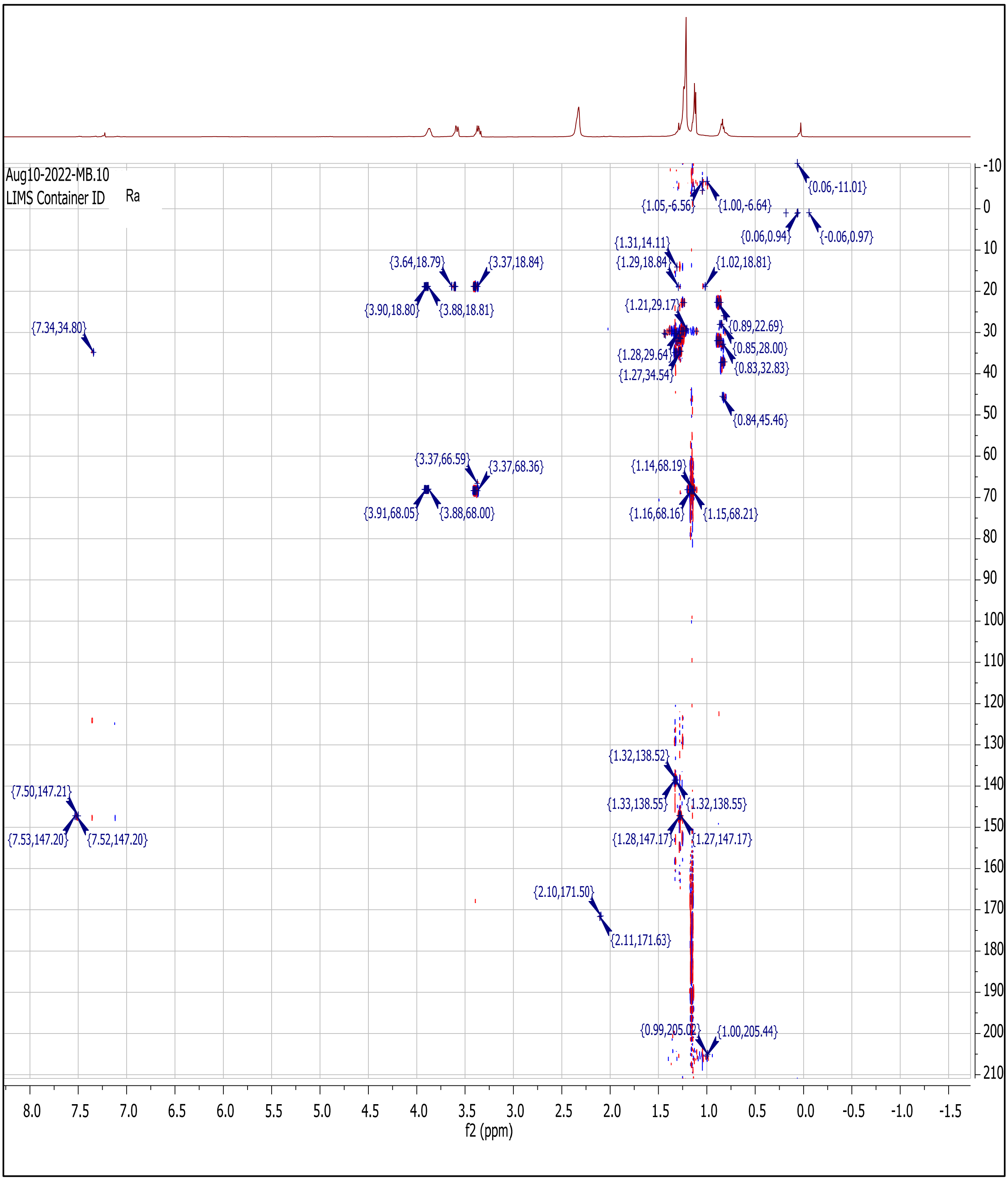

Supplement: Supplementary file 1 [file molecules-28-00899-s001.zip › molecules-2124919-supplementary.pdf]
